# How Body-Centering Improves the Effects of Core Stability Training on the Motor Skills in Adolescent Female Volleyball Players

**DOI:** 10.3390/jfmk10020144

**Published:** 2025-04-25

**Authors:** Arianna Fogliata, Fioretta Silvestri, Lorenzo Marcelli, Maria Chiara Gallotta, Davide Curzi

**Affiliations:** 1 Department of Mental and Physical Health and Preventive Medicine, University of Campania “Luigi Vanvitelli”, 81100 Caserta, Italy; 2 Department of Human Sciences, Pegaso Telematic University, 80143 Naples, Italy; 3 Department of Humanities, Movement and Education Sciences, University “Niccolò Cusano”, 00166 Rome, Italy; fioretta.silvestri@unicusano.it (F.S.);; 4 Department of Physiology and Pharmacology “Vittorio Erspamer”, Sapienza University of Rome, 00185 Rome, Italy

**Keywords:** intra-abdominal pressure, volleyball, female, core stability, motor skills, adolescent, balance, strength, centering, conditioning

## Abstract

**Background**: During growth, the reduction in motor control makes core stability training essential, especially in sports involving dynamic jumps. Given the limited training time of adolescent athletes, finding strategies to maximize the effects of core stability training is crucial. This study analyzed the effects of incorporating body-centering techniques (a method that involves conscious modulation of intra-abdominal pressure to enhance postural stability during motor gestures) into a core stability training protocol on balance, trunk control, and lower limb explosive strength in adolescent volleyball players. **Methods**: Forty-four female volleyball athletes (15.6 ± 1.4 years of age) were randomly divided into three experimental groups: G1 = body-centering + core stability training; G2 = core stability training; and G3 = standard conditioning session. The athletes performed 30 min of differentiated intervention training twice a week for 8 weeks. Balance ability (Berg Balance Scale—BBS and Stork balance stand test—SBST), trunk control (Trunk Control test—TCT), and lower limb explosive strength (broad jump—BJ, squat jump—SJ, and drop jump—DJ) were assessed at the beginning (T0) and the end (T1) of the intervention period, and 12 weeks later (T2). **Results**: Data showed a significant improvement of BBS, SBST, DJ (*p* < 0.01), and TCT (*p* < 0.05) in G1 and G2 at T1 compared to T0, which persisted until T2 except for DJ in both groups. SJ improved only in G1 at T1 compared to T0 (*p* = 0.016). G1 showed a higher rate of improvement in SBST (T1: +18.2%; T2: +16.8%) and in DJ (T1: +3%) compared to G2 (SBST T1: +7.6%, T2: +5.2%; DJ: +2.5%). In addition, only G1 showed a significant improvement rate in BBS score (+2.2%) compared to G3 (+0.4%) at T1. **Conclusions**: These results suggested that core training improves balance, trunk control, and explosive strength in young volleyball athletes with and without body-centering. However, integrating body-centering into core exercises leads to better balance and jumping power than core stability training alone.

## 1. Introduction

Balance, recognized as a fundamental prerequisite for the development of motor skills, is essential for the execution of both precise and functional movements [1,2]. In sports involving jumping actions, such as volleyball, maintaining or quickly recovering a stable body position in space is essential for performance [3]. Targeted balance training can facilitate body control during rapid, complex, or coordinated movements, such as those often required in sports like volleyball [4,5,6]. Volleyball, characterized by various jumping actions with asymmetrical impact or take-off, requires good balance and body weight management, especially of the torso [7], as well as high expressions of explosive strength of the lower limbs [8].

During adolescence, a phase characterized by significant physical changes, the ability to manage the growing body dimensions can be compromised, affecting balance control, movement quality, and sport performance. This period of rapid growth involves changes in height and spatial relationships between body segments, which can have a significant impact on posture and stability [9]. Therefore, motor and sport-specific skills acquired in pre-puberty require adaptation that can significantly influence future performance [10]. Training programs for young athletes, especially in jumping sports, should include exercises aimed at improving body management and balance control, considering individual variations in growth and developmental rates [11].

The effectiveness of core stability (CS) training protocols in this context has been widely demonstrated [12,13,14]. Willardson [15] showed how the integration of CS exercises into training programs significantly improves the basic motor skills of adolescent athletes, supporting their ability to adapt to body changes. CS training exercises increase trunk stability and improve balance, facilitating jumping dynamics [15]. Recent research has revealed how CS protocols lead to improvements in trunk muscular endurance, coordination, and sports performance in adolescents involved in team sports [15,16,17]. A study conducted by Bagherian et al. [18] analyzed the effectiveness of CS exercises in adolescent athletes practicing different sports disciplines, showing improvements in balance and dynamic postural control. Similar benefits were observed by Sharma et al. (2012), who reported improvements in static balance and vertical jump after a 9-week core strengthening program in volleyball players with trunk instability [19]. Furthermore, Monastiridi et al. [20] demonstrated that a core stabilization program improved motor performance and health-related quality of life in adolescents with motor difficulties, highlighting the potential applicability of CS training also in non-athlete youth populations. Given its role in injury prevention and its effects on improving technical execution, CS training is used throughout the sports season, heavily during the pre-season and more distributed throughout the competitive season [21]. Finding strategies to maximize the effects of CS training seems crucial, also given the difficulty of coaching staff to consistently integrate it into their daily training routine.

Intra-abdominal pressure (IAP) modulation plays a key role in core muscle activation, improving posture and balance during complex movements [22]. IAP is defined as the steady-state pressure concealed within the abdominal cavity and resulting from the interaction between the abdominal wall and viscera [23]. IAP regulation depends on the coordination of abdominal muscles, diaphragm, and pelvic floor muscles [24] and contributes to spine and trunk stabilization during movements [25]. This neuromuscular coordination plays a central role in functional movement and athletic performance, particularly in female athletes [26]. Body-centering (BC) is the voluntary and proportional application of IAP to motor gesture acceleration [27,28]. Centering through intra-abdominal pressure modulation remains an underexplored area in sports, traditionally confined to clinical assessments rather than recognized as a potential tool for performance enhancement. A recent systematic review and meta-analysis confirmed that core training significantly improves athletic performance across sports, supporting the rationale for integrating structured core protocols in young athletes’ development [29]. The rationale underlying this research was that young athletes could improve postural control and muscle strength by incorporating BC techniques into CS training. Maximizing the benefits of CS training could help to optimize performance while reducing the risk of injury. To date, no studies have incorporated the BC technique into a core training program or examined its potential benefits for body stability and sport-specific skills. Therefore, this study aimed to analyze the effects of incorporating BC into CS training protocols on balance, trunk control, and lower limb explosive strength in a sample of adolescent female volleyball players. Additionally, a secondary aim was to evaluate the persistence of any effects over time to better understand how to incorporate these sessions into seasonal periodization. We hypothesized that incorporating the BC technique into CS training could lead to greater improvement in balance, trunk control, and lower limb explosive strength of female adolescent volleyball athletes compared to CS training alone or standard conditioning training.

## 2. Materials and Methods

### 2.1. Participants

Forty-four female volleyball athletes (15.6 ± 1.4 years of age), competing in the Elite U18 Italian Youth Volleyball Division (FIPAV-Milan, Italy), participated in this study. All athletes, recruited from Volley Club Segrate 1978 (Segrate, Milan, Italy), were trained and assessed by the same technical and medical staff.

To be eligible for the study, the athletes had to meet the following inclusion criteria:

- To have a non-pathological medical history with no injuries in the six months before the beginning of the research;
- To participate in at least three training sessions per week;
- To have at least three years of elite volleyball training experience competing in the elite youth championships;
- To have a BMI in the normal ranges [30].
- The exclusion criteria were as follows:
- Missing more than 10% of the training sessions during the assessed period;
- Experiencing injuries during the assessed period.

### 2.2. Study Design

**Figure 1:**
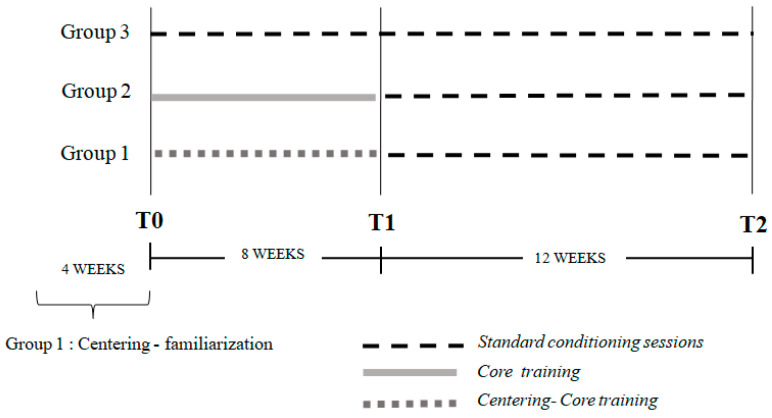
Timeline of the study.

Through simple randomization, the 44 participants were divided into three groups (Group 1: 14 subjects, mean age = 15.5 ± 1.5 years; Group 2: 16 subjects, mean age = 15.7 ± 1.3 years; and Group 3: 14 subjects, mean age = 15.5 ± 1.5 years). Each group followed an individualized training program for 8 weeks (intervention duration). A baseline evaluation of all athletes was performed (T0); moreover, all groups were assessed at the end of the intervention period (T1), and 12 weeks after the end of the intervention period (T2), respectively. The study design timeline of the testing sessions is reported in Figure 1.

### 2.3. Training Protocols

All participants followed volleyball-specific training sessions (2 h for three times a week) and a 1 h conditioning training session twice a week, which included 30 min of differentiated intervention training. The total training volume was the same in all groups.

Before T0, Group 1 carried out 4 weeks of familiarization to learn the BC technique. The sessions included a static phase (aimed at stimulating the conscious use of the IAP from a supine position and in response to external pressure), and a dynamic phase (aimed at using the IAP modulation before performing a requested sport-specific action). The static phase began with supine exercises, in which the athletes voluntarily pushed the abdominal musculature against an external resistance (experimenter’s hand). Subsequently, the quadruped position was introduced, with resistance applied to the paravertebral muscles to stimulate pressure modulation under stable postural conditions. The dynamic phase progressed from a seated to a standing position. In all positions, the progression required the athletes to activate intra-abdominal pressure upon command. The progression was individualized based on execution quality and feedback from the technical team. Body-centering was performed within the core stability exercises and was progressively integrated into each exercise over time. Only during the warm-up phases was centering applied as a separate task, with brief holds of 5–6 s in the first two weeks. This duration was increased by approximately 4 s every two weeks. The progression was adapted according to each subject’s neuromuscular control and execution quality. During the main training, activation was modulated proportionally to the speed and demand of each exercise, with short intra-repetition activations lasting around 3–4 s per repetition. Each session lasted approximately 30 min and involved a dynamic shifting between minimal baseline pressure at rest and increased intra-abdominal pressure during execution. Given the invasiveness of direct IAP measurements, two types of indirect measurements were employed to verify the conscious use of IAP: abdominal circumference measurement [31] and analysis by electronic pressure dynamometer [32]. This screening was carried out by the medical staff in collaboration with the athletic trainer.

During the intervention period (T0–T1), both Group 1 and Group 2 performed a CS training program. However, only Group 1 integrated BC techniques during the CS training exercises. Table 1 shows the CS training exercise protocol with intervals of sets and repetitions; the latter were progressively increased over 8 weeks.

Exercise selection was based on consistency with common practice in youth training and compatibility with the body-centering technique. All exercises were performed using bodyweight only, with no external loads or unstable surfaces, in order to avoid external variables that could affect the impact of the centering technique. Progression was achieved by gradually increasing repetitions and duration over the eight-week intervention.

During the intervention period, Group 3 followed standard conditioning sessions, which included combined cardiovascular and coordinative exercises and flexibility training (focused on shoulder complex). No specific CS or BC+CS training was performed in this group. After the end of the 8-week intervention, all groups followed the same volleyball training program and CS training was suspended in the respective groups. A detailed description of the standard volleyball training program is shown in Table 2.

### 2.4. Motor Skill Measurements

At T0, T1, and T2, all subjects were evaluated in the following areas: balance, trunk control, and lower limb explosive strength. Before T0, all athletes carried out two fitness test familiarization sessions.

Balance assessment:

- Berg Balance Scale (BBS):

The BBS assesses both dynamic and static balance and consists of 14 different tasks involving activities such as stepping, sitting, standing, or transferring body weight in different positions. Athletes’ body control and balance capabilities were evaluated with a rating scale ranging from 0 (unable to perform the task) to 4 (excellent execution of task performance) [33].

- Stork balance stand test (SBST):

The SBST is used to assess static balance. The subject is asked to stand on one foot, lifting the other foot and placing it against the knee of the supporting leg, maintaining this position as long as possible. The test finishes when the subject loses balance or when the lifted foot loses contact with the contralateral knee. Performance is measured in seconds [34].

Trunk control assessment:

- Trunk Control test (TCT):

The TCT involves a series of trunk movements, including flexion, extension, lateral tilting, and rotation, performed from a sitting position with flexed knees at approximately 90-degree angle and fixed arms (at shoulders or chest, depending on the subject’s preference) [35]. Given that body-centering training involves multi-directional intra-abdominal pressure modulation, the TCT was selected for its ability to assess trunk control across multiple planes. Its structure, including flexion, extension, lateral tilts, and rotation, offers a comprehensive functional profile of trunk stability, aligning with the multidimensional nature of the intervention. Additionally, the seated posture was chosen to minimize compensatory strategies from the lower limbs and to isolate trunk-specific responses, allowing for a focused observation of core activation. For this reason, and due to the practical requirements of youth sports settings, the TCT was preferred over other more commonly used sport-specific tests, for example, the Star Excursion Balance Test (SEBT) or the isometric core endurance protocol proposed by McGill [36,37]. Specific instructions were provided for each movement, emphasizing the importance of maintaining the position as stable as possible during execution (30 s). The level of stability and body control during movement execution was evaluated on a scale from 0 to 3 (Table 3).

Lower limb explosive strength assessment

- Broad jump test (BJ):

Athletes are asked to stand behind a starting line with feet about shoulder-width apart. From this position, the athlete has to bend the knees (self-selected flexion depth) and jump forward as far as possible, landing on both feet in a controlled fashion. The outcome is the average distance (cm), from the starting line to the nearest heel landing point, covered in three valid attempts performed (resting time of 90 s between attempts) [38].

- Squat jump (SJ) and drop jump (DJ) tests:

The two tests are performed using a force platform to estimate jumping variables (Contact Platform, Chronojump Bosco System, Barcelona, Spain) [39]. For both tests, a total of 6 attempts are performed, distributed in three sets of two jumps per jump type. The best outcomes score over all attempts is taken for further analyses.

In the SJ, athletes are asked to perform a maximal vertical jump starting from a static squatting position, with knees bent at about a 90-degree angle and blocked arms along the sides to prevent any arm contribution to propulsion. In the DJ, the athlete is asked to jump down from a raised platform (40 cm), landing on both feet on the force platform, and immediately perform a maximal vertical jump. The goal is to minimize the ground contact time and maximize the height of the subsequent jump, to transform the kinetic energy of impact into explosive energy for the jump [40]. The outcome of SJ and DJ tests is the jump’s power reached in the attempts [41].

### 2.5. Statistical Analysis

Test scores of the three groups were analyzed using IBM SPSS statistics 25.0 software. In the analysis of data distribution, the Shapiro–Wilk test showed significant deviations from normality in BBS, TCT, SJ, and DJ variables and thus, non-parametric analysis was used for data processing. The Friedman Test for non-parametric analysis of variance was used to detect differences among groups and a post hoc analysis was conducted using the Wilcoxon signed-rank test to examine specific differences between the time pairs within the same group. Moreover, for each significantly improved test, we calculated the percentage of variation with respect to its pre-intervention values (T1−T0 and T2−T0). The Kruskal–Wallis test was then performed to examine the effect of the type of training (Group 1 vs. Group 2 vs. Group 3) on the percentage of variation. Additionally, the Mann–Whitney test was used to allow group paired comparisons when significant changes were detected. For the within-group analysis, the post hoc GPower reported a statistical power (=1 − β) of 0.82 with a large effect size (d = 0.5) for the sample size of the groups included in the study. In the between-group analysis, considering the total sample size, the post hoc GPower reported a power (=1 − β) of 0.85 for a large effect size (d = 0.45). Significance was set at *p* ≤ 0.05.

## 3. Results

### 3.1. Pre–Post-Intervention

The Friedman Test showed significant differences among groups (*p* < 0.001) through the intervention period (T0 vs. T1). Specifically, Group 1 showed significant improvements in BBS (*p* = 0.005), SBST (*p* < 0.001), TCT (*p* = 0.02), SJ (*p* = 0.016), and DJ (*p* = 0.001) at T1 compared to T0. Group 2 also showed significant improvements in BBS (*p* = 0.003), SBST (*p* = 0.001), TCT (*p* = 0.014), and DJ (*p* = 0.0001) after intervention (T0 vs. T1). These results showed an improvement in the outcomes of numerous tests in Groups 1 and 2 at the end of the intervention period, while no significant changes were found in Group 3.

### 3.2. Follow-Up Analysis

Follow-up analysis showed that improvements in several tests persisted after the end of the intervention period compared to baseline values. At T2, Group 1 still showed significant improvement in some previously enhanced tests, compared to T0 (BBS: *p* = 0.03; TCT: *p* = 0.04; SBST: *p* < 0.01;). In Group 2, significant improvements were still observed in BBS (*p* = 0.011), TCT (*p* = 0.044), and SBST (*p* = 0.002) at T2, compared to T0. These results reveal that significant improvement mainly persisted in Groups 1 and 2 at the follow-up. Average values from T0 to T2 of all groups were reported in Table 4.

### 3.3. Delta Scores Group Comparisons

**Figure 2:**
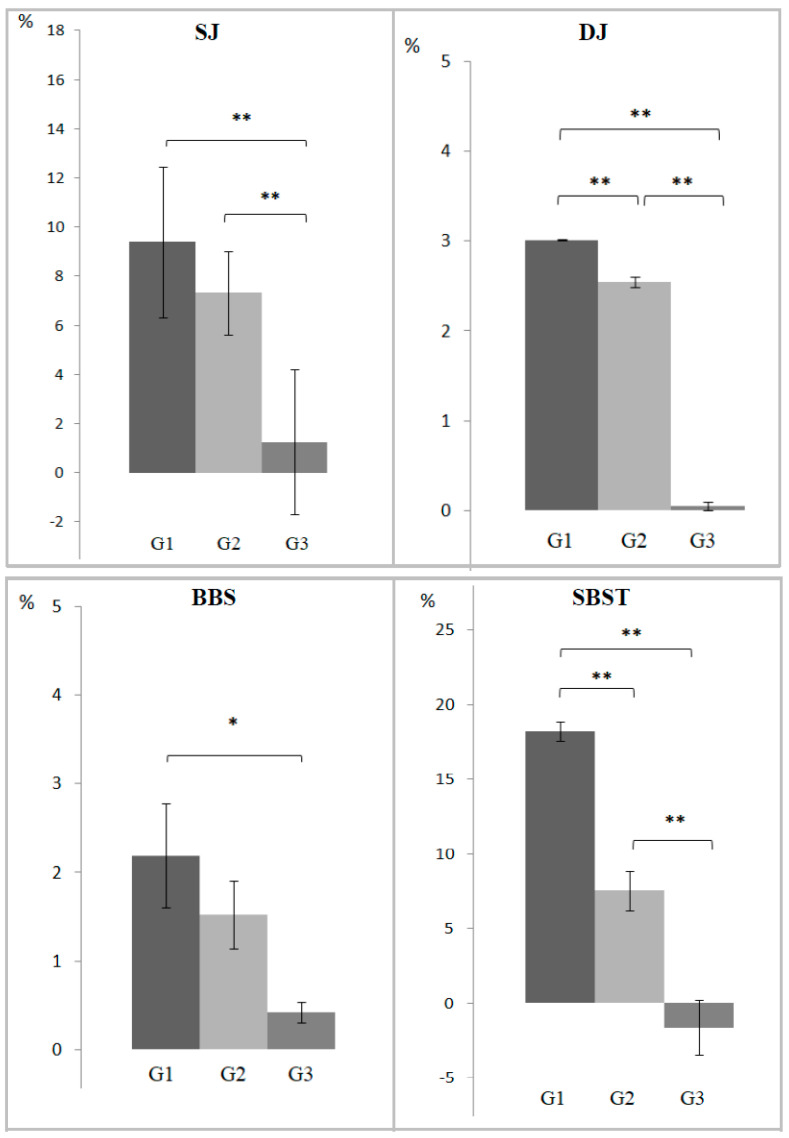
The y axis represents the percentage of variation (post- to pre-intervention: T1−T0) of the significant tests in the three groups. The values are reported as mean and standard error. * = *p* < 0.05 significant difference between groups; ** = *p* < 0.01 significant difference between groups. Abbreviations: BBS, Berg Balance Scale; SBST, Stork balance stand test; SJ, squat jump test; DJ, drop jump test. G1 = Group 1; G2 = Group 2; and G3 = Group 3.

Kruskal–Wallis analysis of percentage of variation (T1 vs. T0) revealed significant differences in four out of six tests (BBS, SBST, SJ, and DJ) among groups. Post hoc analysis between groups, pairwise, showed a higher improvement rate of SBST and DJ (*p* < 0.01) in Group 1 (+18.2% and +3%) compared to Group 2 (+7.6% and +2.5%), of BBS (*p* = 0.035) in Group 1 (+2.2%) compared to Group 3 (+0.4%), and in SBST, SJ, and DJ (*p* < 0.01) in Group 1 (+18.2%; +9.4% and +3%) compared to Group 3 (−1.6%; +1.3 and +0.1%). In addition, Group 2 showed a better improvement rate in SBST, SJ, and DJ (*p* < 0.01) compared to Group 3 (% values above). Finally, Group 1 showed a significantly higher improvement rate in SBST (+16.8%) from T0 to T2 compared to Group 2 (+5.2%) (*p* < 0.01), although both groups still maintained a significant improvement compared to Group 3 (−3.4%) (*p* < 0.01). The results of delta scores (T1−T0) of the tests with significant differences between groups are represented in Figure 2 and follow-up data (T2−T0) are reported as Supplementary Materials (Figure S1).

## 4. Discussion

This study investigated the effects of incorporating BC techniques into a CS training protocol on balance ability, trunk control, and lower limb explosive strength in a sample of adolescent female volleyball players. The main finding of this study suggested an improvement in balance ability and jumping performance when BC techniques are integrated into CS training, allowing athletes to obtain better results from CS training without increasing CS training time. In light of these findings, it was possible to hypothesize that this improvement could indirectly induce a technical and athletic performance upgrade. These positive effects could be due to the synergistic activation of the abdominal muscles, through the voluntary flattening of the thoracic diaphragm, induced by the technical BC [28].

### 4.1. Balance

Significant improvements in BBS scores were found after the intervention period in Groups 1 and 2, while no improvements were observed in Group 3 (control group). These results suggested that CS training could improve balance performance in young volleyball athletes, as reported in the literature [42,43]. Similar results on CS training were reported by Oliver and Di Brezzo [44], who demonstrated that stability exercises could significantly improve balance performance in athletes, and by Kavanaugh et al. [45], who highlighted the role of core strength in improving overall athletic performance in a long-term period. However, BC integration in CS training can further amplify these benefits. Indeed, Group 1, but not Group 2, showed a significant BBS increase compared to Group 3 at the end of the intervention period.

Similarly, Groups 1 and 2 showed significant improvements in the Stork balance test from T0 to T1, in contrast to Group 3. In agreement with previous studies, these results demonstrated the effectiveness of CS exercises in improving athletes’ balance [15,20]. The integration of BC techniques into CS training further amplified these benefits. Athletes who performed this training showed a higher rate of improvement than those who followed only CS training (*p* < 0.01). Furthermore, although at follow-up both groups (1 and 2) showed significant increases in SBST scores compared to T0, Group 1 maintained a significantly higher level of SBST performance at follow-up compared to Group 2 (*p* < 0.01). This additional benefit in balance performance observed in athletes who followed BC+CS training could be due to both the increased activation of deep trunk muscles and the resulting improvement in body awareness [46]. These results suggest that integrating BC techniques into CS training may lead to more durable and stable improvements in balance compared to CS training alone.

### 4.2. Trunk Control

A significant improvement in trunk control was found in athletes of Groups 1 and 2 in contrast to those from Group 3. These results were in line with the published literature, which showed that both CS training and BC techniques improved body control and trunk strength in young athletes [47,48]. In agreement with these results, Dewi and Palgunadi [49] highlighted how CS training could improve trunk control and postural stability, while Dong et al. [50] demonstrated through a meta-analysis the significant role of core strengthening in increasing trunk support strength in various sporting contexts. Furthermore, a randomized controlled trial by Granacher et al. (2014) confirmed similar benefits, highlighting the importance of CS training, both on stable and unstable surfaces, in improving athletes’ functional abilities and general physical fitness in adolescents [51]. Reed et al. [52] also confirmed the importance of CS training in improving balance and athletic performance through a comprehensive review of the literature. Furthermore, in line with the results on balance, high and significant levels of trunk control were found also at follow-up in both experimental groups. These results, in agreement with previous research, suggested the key role that CS training could play in those sports activities characterized by plyometric and vertical jump performances [19,53]. As a potential functional activator of the core area, BC techniques could play a role in trunk control strategies; however, further investigations are needed to fully understand their potential and effectiveness in the sports context.

### 4.3. Jump Performance

Group 1 showed a significant improvement in SJ score at the end of the intervention, while no significant changes were found in the other two groups. The ability to voluntarily activate and modulate the IAP during static exercises could facilitate the transfer to sport-specific movements, such as jumping, through increased body awareness.

The DJ test showed significant improvements between T0 and T1 in both experimental groups (Groups 1 and 2), while no changes were observed in Group 3. Furthermore, the improvements observed in Group 1 were significantly higher than in Group 2. The DJ, which involves a more complex movement of the SJ, would require maximum use of the BC in an already dynamic phase, requiring a rapid transition between ground contact and jump. In agreement with these results, Hoshikawa and colleagues found significant improvements in jumping performance (SJ and CMJ) after six months of a trunk stability program in young soccer players [54]. With a study design similar to this research, Ferri-Caruana and colleagues assessed the effects of an eight-week core training program (two times per week) on adolescent female handball players [55]. The authors, according to the results of this study, found a significant increase in DJ performance after the end of the intervention. However, in the both cited studies, the authors did not assess the IAP within the intervention, which could have played a significant role based on our results. In fact, previous studies have observed higher IAP for the DJ than for vertical jumping and increasing pressure with increasing load [56]. Thus, the use of BC could be particularly advantageous in motor activities that require expressions of force associated with direction changes, such as in the DJ. In summary, the effectiveness of the BC may be particularly relevant for jumping exercises that require static and dynamic control before or during the execution of the movement. These findings are also consistent with the results of a recent systematic review, which confirmed that trunk muscle training improves both general physical fitness and sport-specific performance in young and adult athletes [57]. These findings suggest that integrating trunk activation strategies, such as body-centering, may enhance neuromuscular efficiency and explosive performance in young athletes. Further studies are needed to understand how modulation of intra-abdominal pressure contributes to reactive force development and the efficiency of the stretch-shortening cycle. Although our study showed significant improvements in both squat jump (SJ) and drop jump (DJ) after the intervention, the follow-up data (T2) suggest that the retention of improvements in DJ is less stable. This may be due to the higher neuromuscular complexity required by the DJ, which involves a rapid transition between impact absorption and explosive push-off.

While the body-centering technique may support core activation in more controlled movements like the squat jump, its effect on more reactive and dynamic actions might require a longer consolidation period or additional specific training. This aspect warrants further investigation.

### 4.4. Limitations of the Study

The number of athletes involved was limited to ensure that the technical and medical staff were the same for all athletes and that the seasonal work was comparable.

The sample, uniform in terms of age distribution and ability level, limits the result to extension to other contexts. Moreover, with the included sample size, only large differences between groups were observed, since smaller differences would necessitate a larger sample for detection.

Another limitation of the present study is the lack of male volleyball players, highlighting the necessity for further research among the male population to determine whether the findings are consistent with or differ from those observed in the female population of this study. An indirect measurement of IAP was preferred to direct measurement techniques, although the latter provided more reliable results. Direct measurements require invasive methods, often used in clinical settings, but are difficult to practice in a sport environment on adolescent subjects. Furthermore, more dynamic balance tests, such as the Star Excursion Balance Test (SEBT) or the Y Balance Test, could have offered deeper insights into the study results, as they are better suited for the athletic population. Finally, although the follow-up results suggest a maintenance of the effects, we acknowledge that other training components (e.g., sport-specific technical work) may have contributed to performance stability, although likely to a minimal extent, since all groups followed the same technical program after the intervention. Therefore, we cannot completely exclude the influence of external factors.

## 5. Conclusions

This study explored the effects of integrating body-centering into core strength training in young, competitive female volleyball players. The findings showed that adding body-centering led to significant improvements in balance, trunk control, and jump performance, with results of greater magnitude than core strength training alone or standard conditioning. Moreover, the integration of body-centering techniques into core strength training showed a positive long-lasting effect extending far beyond the intervention period, with greater effects on balance ability observed in the group using integrated training compared to core strength training alone. The body-centering technique can be integrated into youth training programs, optimizing athletic performance without increasing training loads. Furthermore, this technique could be integrated into existing training routines without requiring dedicated time, finding practical applications in sporting contexts where time is increasingly limited. Further studies should focus on the ability to incorporate body-centering technique into sport-specific technical gestures that go beyond the “stop-and-go” movements used in this research protocol. Understanding the effects of body-centering on additional sport-specific skills could open new fields of investigation for performance optimization in young athletes.

## Figures and Tables

**Table 1:** CS training protocol exercises.

Exercise	Sets	Reps
Plank	3–5	30–60 s
Bird-dog	3–5	10–12 reps
Russian Twist	3–5	12–16 reps per side
Leg Raises	3–5	10–12 reps
Dead Bug	3–5	8–12 reps per side
Side Plank (both sides)	3–5	30–45 s per side
Leg Bridge	3–5	10–20 reps

**Table 2:** Description of the volleyball standard conditioning program.

Period	Type of Exercise	Specific Activities	Progression
Weeks 1–2 G3	Aerobic (10′) Footwork drills (6′) Lower limb activation (6′) General mobility (8′)	- Steady-pace running - Basic locomotor drills - Static calf raises - Mobility for neck, shoulders, wrists, and fingers	Low-intensity activation. Focus on joint range of motion and basic motor control.
Weeks 3–4 G3	Aerobic (10′) Footwork drills (6′) Lower limb activation (6′) General mobility (8′)	- Running with light directional changes - Alternating movement patterns - Dynamic calf raises - Broader range mobility exercises	Increased movement variability and amplitude. Slight rise in motor complexity.
Weeks 5–6 G3	Aerobic (10′) Footwork drills (6′) Lower limb activation (6′) General mobility (8′)	- Running with tempo variations - Combined, faster drills - Calf raises with isometric holds - Fluid mobility sequences	Introduction of rhythm changes and enhanced neuromuscular control.
Weeks 7–8 G3	Aerobic (10′) Footwork drills (6′) Lower limb activation (6′) General mobility (8′)	- Running and direction shifts - Articulated movement sequences - Advanced calf raise variations - Full-body joint mobility routines	Peak phase of dynamic work. Emphasis on coordinated multi-joint control.
Follow-up (G1–G2–G3)	General physical maintenance	- Cardiovascular drills and footwork-based movement games - Muscle work targeting shoulder and lower limb chains	No core stability or body-centering exercises included.

**Table 3:** Trunk Control test description.

Movement	Description	Time	Score Criteria
Flexion	Lean forward from the trunk while keeping the back straight	30″	0 = The individual is unable to perform the movement on their own. 1 = The individual is able to perform the movement with some difficulty or instability. 2 = The individual is able to perform the movement with minimal difficulty. 3 = The individual is able to perform the movement perfectly and without difficulty.
Extension	Arch the back backward while keeping the trunk stable	30″	
Lateral inclination	Tilt the trunk laterally from side to side	30″	
Rotation	Rotate the trunk from side to side	30″	

**Table 4:** Motor skill test results from T0 to T2.

	Group 1-T0	Group 1-T1	Group 1-T2
	Mean ± St-Dev	Mean ± St-Dev	Mean ± St-Dev
BBS [score]	39.93 ± 1.82	40.79 ± 1.67 **	40.71 ± 1.64 *
TCT [score]	9.36 ± 1.15	9.79 ± 1.37 *	9.71 ± 1.20 *
BJT [cm]	243.21 ± 18.77	244.29 ± 18.69	241.43 ± 19.16
SBST [s]	11.14 ± 1.46	13.14 ± 1.46 **	13.00 ± 1.57 **
SJ [watt]	400.60 ± 54.84	436.36 ± 65.22 *	400.27 ± 21.29
DJ [watt]	36,562.14 ± 11,169.28	37,663.79 ± 11,506.14 **	36,552.14 ± 11,169.29
	**Group 2-T0**	**Group 2-T1**	**Group 2-T2**
	Mean ± St-Dev	Mean ± St-Dev	Mean ± St-Dev
BBS [score]	38.06 ± 2.35	38.63 ± 2.13 **	38.38 ± 2.33 *
TCT [score]	8.44 ± 0.89	8.81 ± 1.05 *	8.81 ± 1.11 *
BJT [cm]	231.56 ± 16.71	232.50 ± 16.53	231.25 ± 15.86
SBST [s]	9.00 ± 1.15	9.69 ± 1.35 **	9.44 ± 1.15 **
SJ [watt]	394.17 ± 44.71	422.17 ± 46.38	388.04 ± 42.84
DJ [watt]	41,747.48 ± 1176.17	42,829.49 ± 1254.90 **	41,737.48 ± 1176.17
	**Group 3-T0**	**Group 3-T1**	**Group 3-T2**
	Mean ± St-Dev	Mean ± St-Dev	Mean ± St-Dev
BBS [score]	36.93 ± 2.16	37.07 ± 2.02	37.00 ± 2.35
TCT [score]	9.07 ± 0.83	9.14 ± 1.35	8.64 ± 1.55
BJT [cm]	235.36 ± 18.02	236.43 ± 14.47	235.00 ± 10.92
SBST [s]	10.14 ± 2.68	9.93 ± 2.62	9.79 ± 2.61
SJ [watt]	381.11 ± 58.02	382.19 ± 47.82	389.44 ± 47.69
DJ [watt]	42,844.05 ± 4883.80	42,866.37 ± 4879.78	42,929.83 ± 4840.96

Significantly improved values vs. T0 within the same group are in bold: * *p* < 0.05; ** *p* < 0.01. Abbreviations: BBS, Berg Balance Scale; TCT, Trunk Control test; BJ, broad jump test; SBST, Stork balance stand test; SJ, squat jump test; and DJ, drop jump test.

## Data Availability

The raw data that support the findings of this study are available from the corresponding author, M.C.G., upon reasonable request due to the informed consent agreement, which does not account for public sharing of the data.

## Supplementary Materials

The following supporting information can be downloaded at: https://www.mdpi.com/article/10.3390/jfmk10020144/s1, Figure S1. Percentage of variation at follow-up (12 weeks post-intervention to pre-intervention) of the three groups.
