# Supplementary material for: How Body-Centering Improves the Effects of Core Stability Training on the Motor Skills in Adolescent Female Volleyball Players
Source: J Funct Morphol Kinesiol. 2025 Apr 25;10(2):144. doi: 10.3390/jfmk10020144 (PMC12101249; doi:10.3390/jfmk10020144)
Supplement: Supplementary file 1 [file jfmk-10-00144-s001.zip › jfmk-3496887-supplementary.pdf]

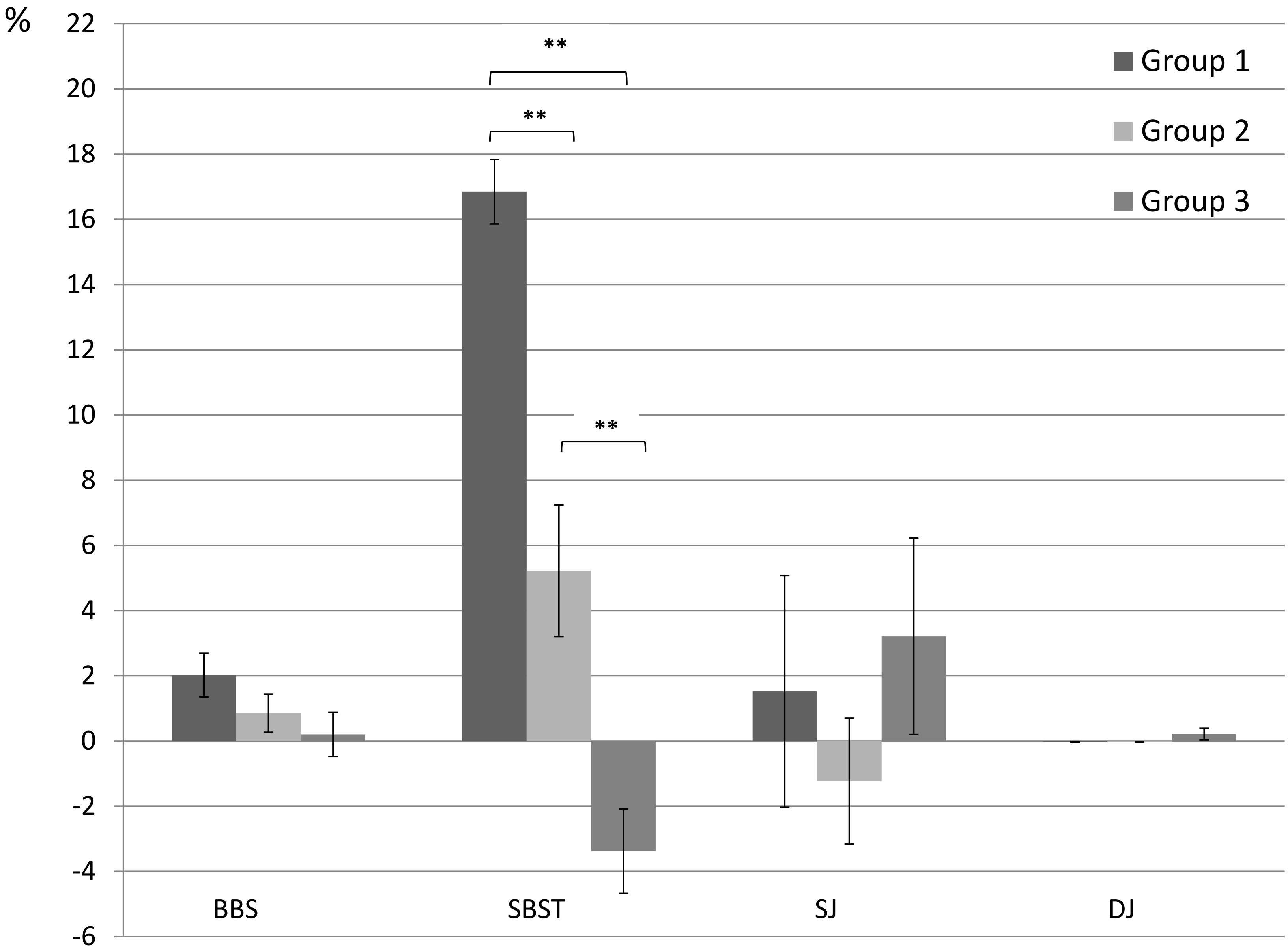

Figure S1. Percentage of variation at follow-up (12 weeks post-intervention to pre-intervention) of the three groups. Abbreviations: BBS, Berg Balance Scale; SBST, Stork balance stand test; SJ, Squat Jump test; DJ, Drop Jump test. * $p < 0.05$; ** $p < 0.01$.
